# Supplementary material for: Method for assessing the spatiotemporal resolution of structured illumination microscopy (SIM)
Source: Biomed Opt Express. 2021 Jan 12;12(2):790–801. doi: 10.1364/BOE.403592 (PMC7901338; doi:10.1364/BOE.403592)
Supplement: Supplementary file 1 [file boe-12-2-790-s001.pdf]

Supplemental Document

Biomedical Optics EXPRESS

Method for assessing the spatiotemporal resolution of structured illumination microscopy (SIM): supplement

ABDERRAHIM BOUALAM AND CHRISTOPHER J. ROWLANDS*

Department of Bioengineering, Imperial College London, South Kensington, London, SW7 2BP, UK
**c.rowlands@imperial.ac.uk*

This supplement published with The Optical Society on 12 January 2021 by The Authors under the terms of the Creative Commons Attribution 4.0 License in the format provided by the authors and unedited. Further distribution of this work must maintain attribution to the author(s) and the published article's title, journal citation, and DOI.

Supplement DOI: https://doi.org/10.6084/m9.figshare.13473759

Parent Article DOI: https://doi.org/10.1364/BOE.403592

A method for assessing the spatiotemporal resolution of Structured Illumination Microscopy (SIM): supplemental document

ABDERRAHIM BOUALAM[1] AND CHRISTOPHER J ROWLANDS[1,*]

[1]*Department of Bioengineering, Imperial College London, South Kensington, London, SW7 2BP, UK*
**c.rowlands@imperial.ac.uk*

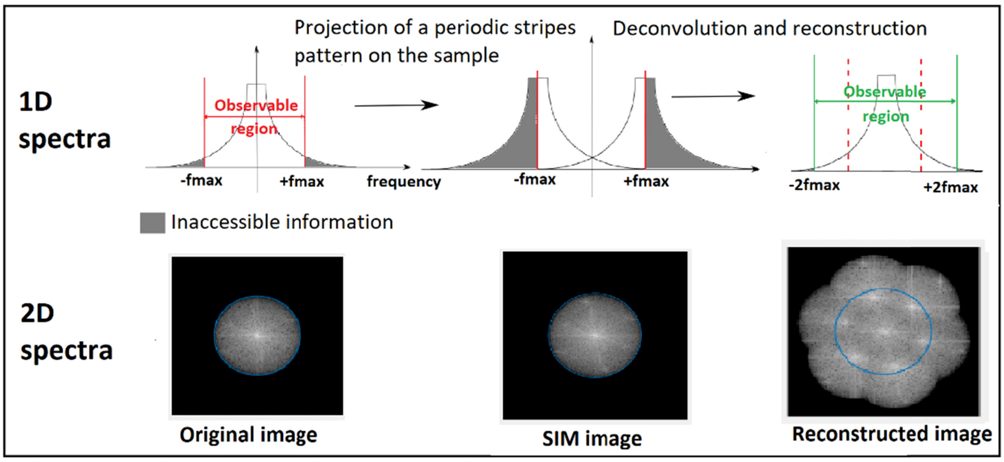

Fig. S1: SIM resolution enhancement, visualized in the Fourier domain. The upper row shows a 1D illustration of the Optical Transfer Function, whereas the lower row plots the magnitude of the Fourier Transform for an illustrative sample. Left: spectra of a widefield image. Middle: spectra of the widefield image illuminated with a striped pattern. Right: spectra of a high-resolution SIM reconstructed image. SIM imaging gives access to an observable region approximately twice as large as that of widefield image (blue circle). Modified from *(3)*.

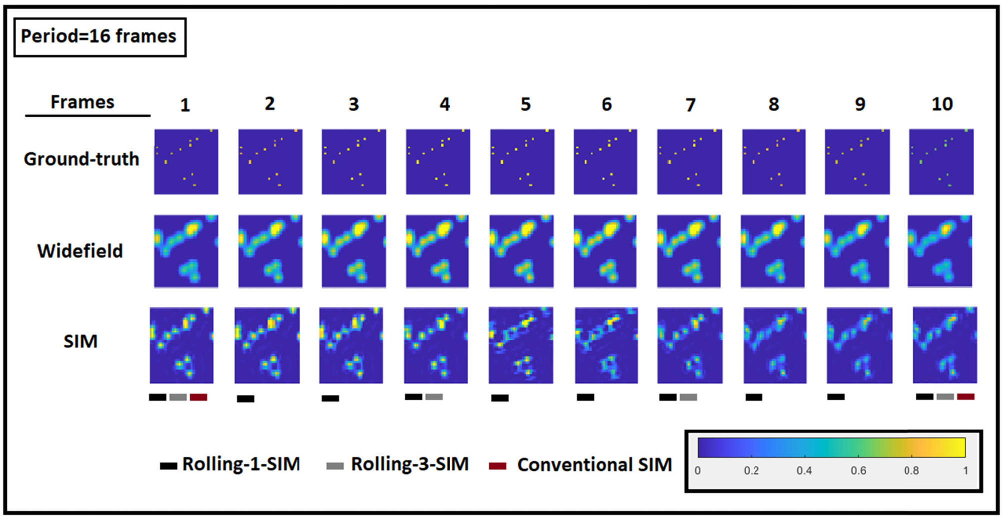

Fig. S2: Illustration of the imaging performance of SIM reconstruction in the presence of pronounced high-temporal-frequency oscillations. In an ideal case, all reconstructed frames would be identical in appearance, because the SIM reconstruction would not be affected by temporal modulation.

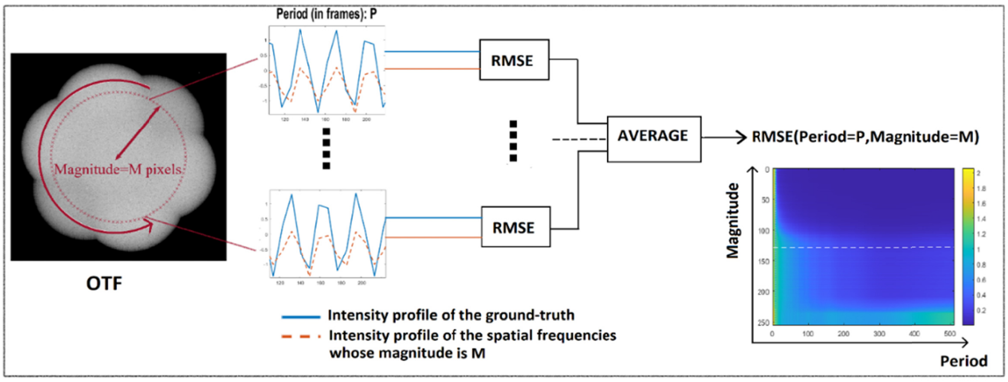

Fig. S3: Calculation of the RMSE metric. RMSE between the ground-truth intensity profile and that of spatial frequencies with the same magnitude M are averaged radially (see the red circular arrow on the OTF) and plotted for different modulation periods P (see right hand side plot)

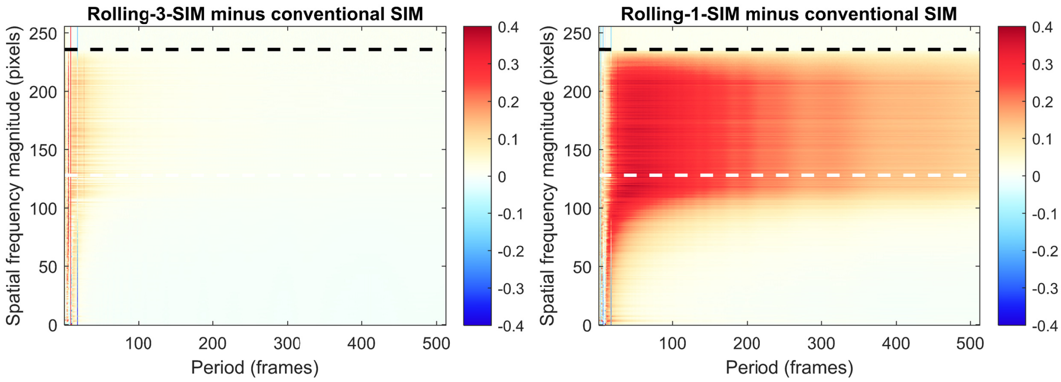

Fig. S4: Difference between the temporal resolution of Rolling-3-SIM and conventional SIM, and of Rolling-1-SIM and conventional SIM, obtained by subtracting the RMSE for conventional SIM from that of Rolling SIM; data displayed with identical colour scales. The white and black horizontal dashed lines highlight the theoretical diffraction limit and the limit of the SIM OTF respectively.
